# Supplementary material for: Spinal Muscular Atrophy after Nusinersen Therapy: Improved Physiology in Pediatric Patients with No Significant Change in Urine, Serum, and Liquor 1H-NMR Metabolomes in Comparison to an Age-Matched, Healthy Cohort
Source: Metabolites. 2021 Mar 30;11(4):206. doi: 10.3390/metabo11040206 (PMC8065886; doi:10.3390/metabo11040206)
Supplement: Supplementary file 1 [file metabolites-11-00206-s001.pdf]

Electronic supplementary material

Spinal muscular atrophy after nusinersen therapy: improved physiology in pediatric patients with no significant change in urine, serum and liquor 1H-NMR metabolomes in comparison to an age-matched, healthy cohort

Leon Deutsch [1], Damjan Osredkar [2, 3], Janez Plavec [4] and Blaž Stres [1, 5,6,7*]

[1] University of Ljubljana, Biotechnical Faculty, Department of Animal Science, Ljubljana, Slovenia; leon.deutsch@bf.uni-lj.si

[2] Department of Pediatric Neurology, University Children's Hospital, University Medical Centre Ljubljana, Slovenia; damjan.osredkar@kclj.si

[3] Faculty of Medicine, University of Ljubljana, Slovenia

[4] National Institute of Chemistry, NMR Center, Ljubljana, Slovenia; janez.plavec@ki.si

[5] Jožef Stefan Institute, Department of Automation, Biocybernetics and Robotics, Ljubljana, Slovenia

[6] University of Ljubljana, Faculty of Civil and Geodetic Engineering, Institute of Sanitary Engineering, Ljubljana, Slovenia

[7] University of Innsbruck, Department of Microbiology, Innsbruck, Austria

* Correspondence: blaz.stres@bf.uni-lj.si, Tel.: +38641567633

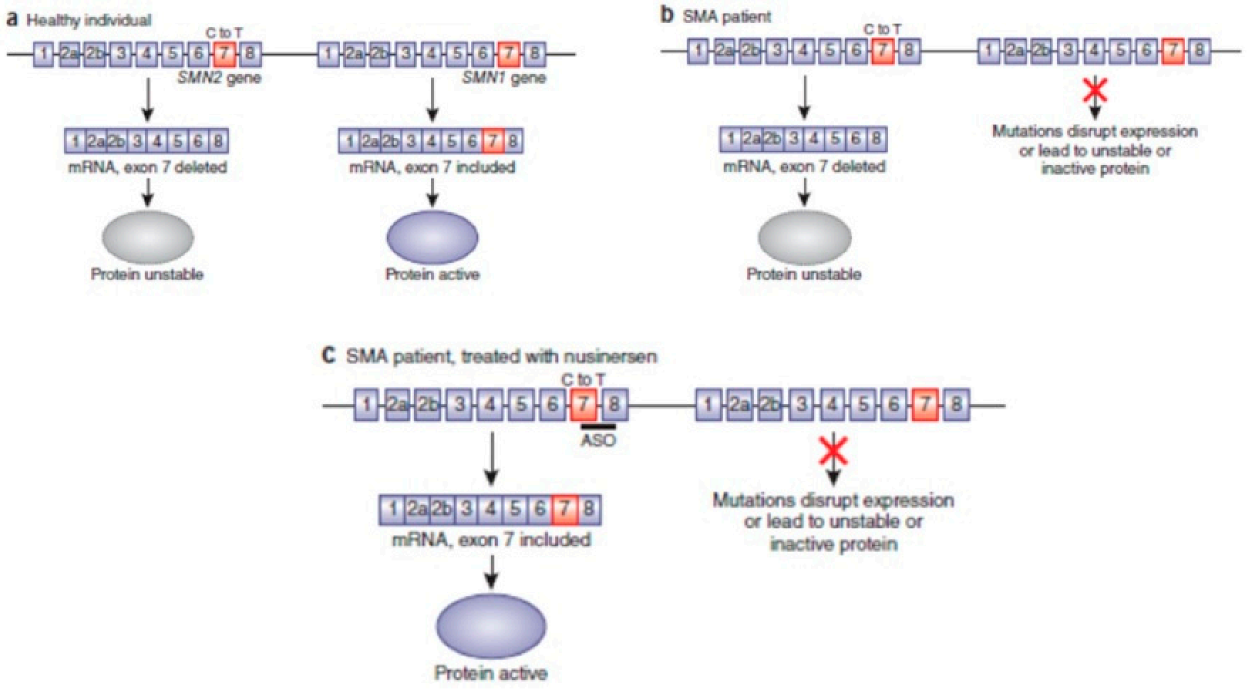

Figure S1. Healthy individuals have active protein SMN1 (a). In SMA patients mutations leads to expression of unstable protein SMN1. Because of that unstable protein expressed by SMN2 gene leads to manifestation of SMA disease (b). Treatment with nusinersen increased the proportion of active protein with inclusion of exon 7 into SMN2 gene, which leads to the improvement of the disease [14].

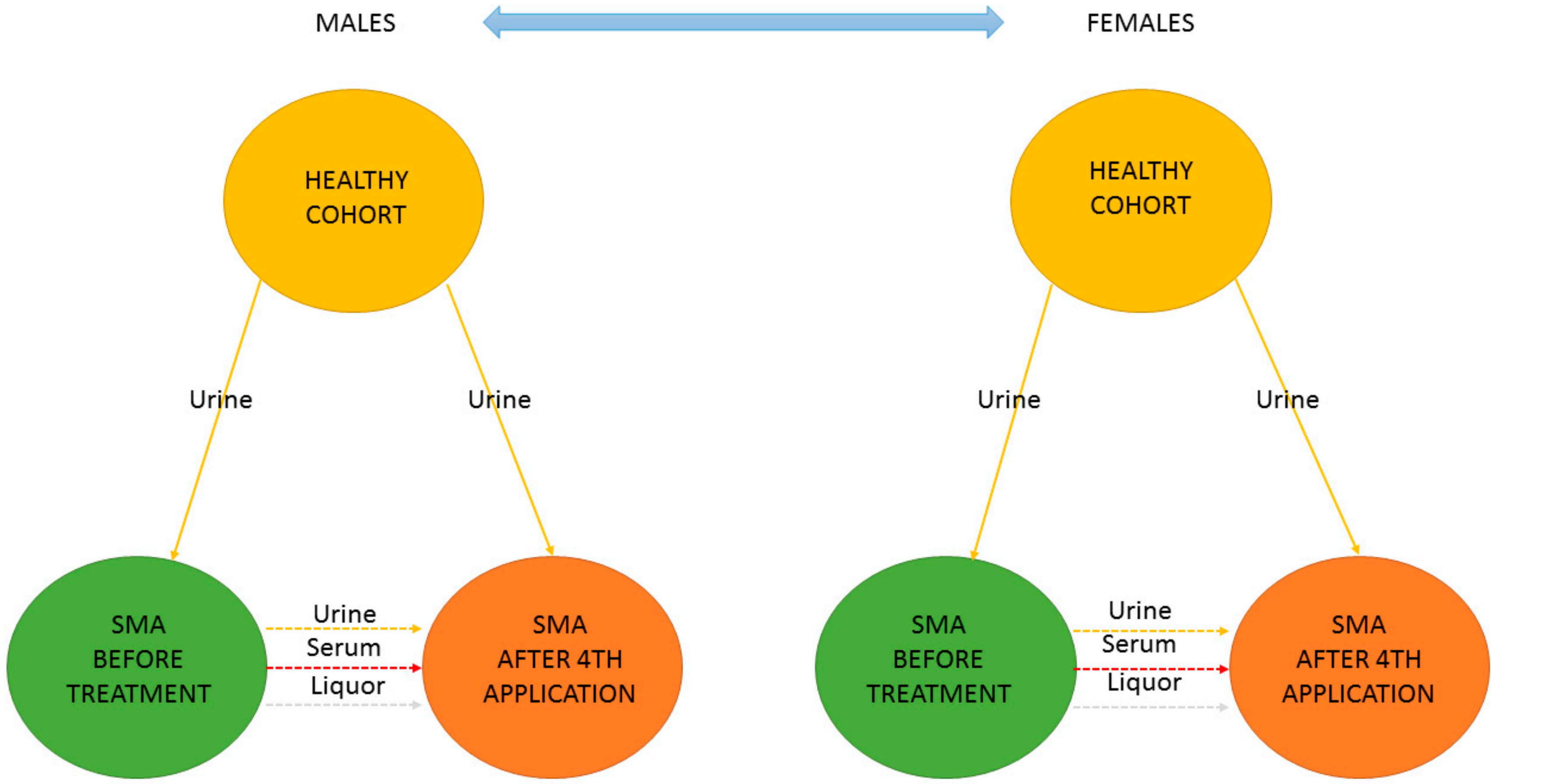

Figure S2. Sample collection and data analysis scheme. Samples were collected for SMA patients before and after 4th application of nusinersen and are coded by the color of the arrow: urine (yellow), liquor (grey) and serum (red). Urine samples from age- and sex- matched healthy cohorts (yellow) were also collected for additional comparisons. The arrows designate also the relationships tested between the groups of collected samples using the ensemble of MetaboAnalyst, npMANOVA and JADBIO approaches.

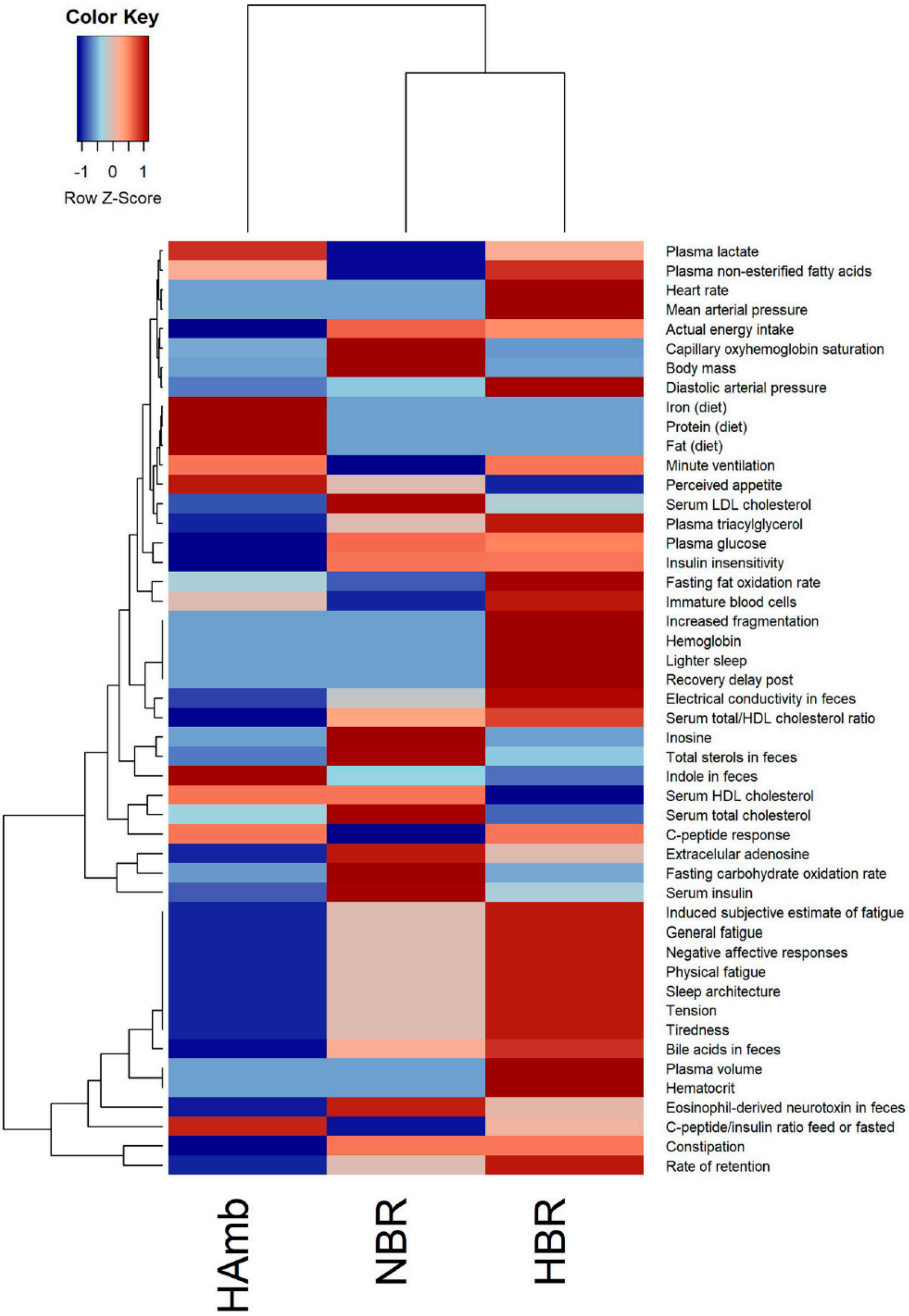

Figure S3: Heatmap plot showing the relationship between parameters describing human physiology, psychology, and intestinal environment that differed significantly at the end of the PlanHab experiment ($n = 48$; $p < 0.05$; FDR corrected) that are now part of the new version of the in-house PlanHab database (Sket et al., 2017b) based on all measured variables within the project. The inset to the left reresents the magnitude of z-normalized data.

(Reproduced from Sket et al., 2020. Authors publishing with Frontiers have their articles under the Creative Commons licence CC-BY which permits re-use, distribution and reproduction of material from published articles, provided the original authors and source are credited [2]).

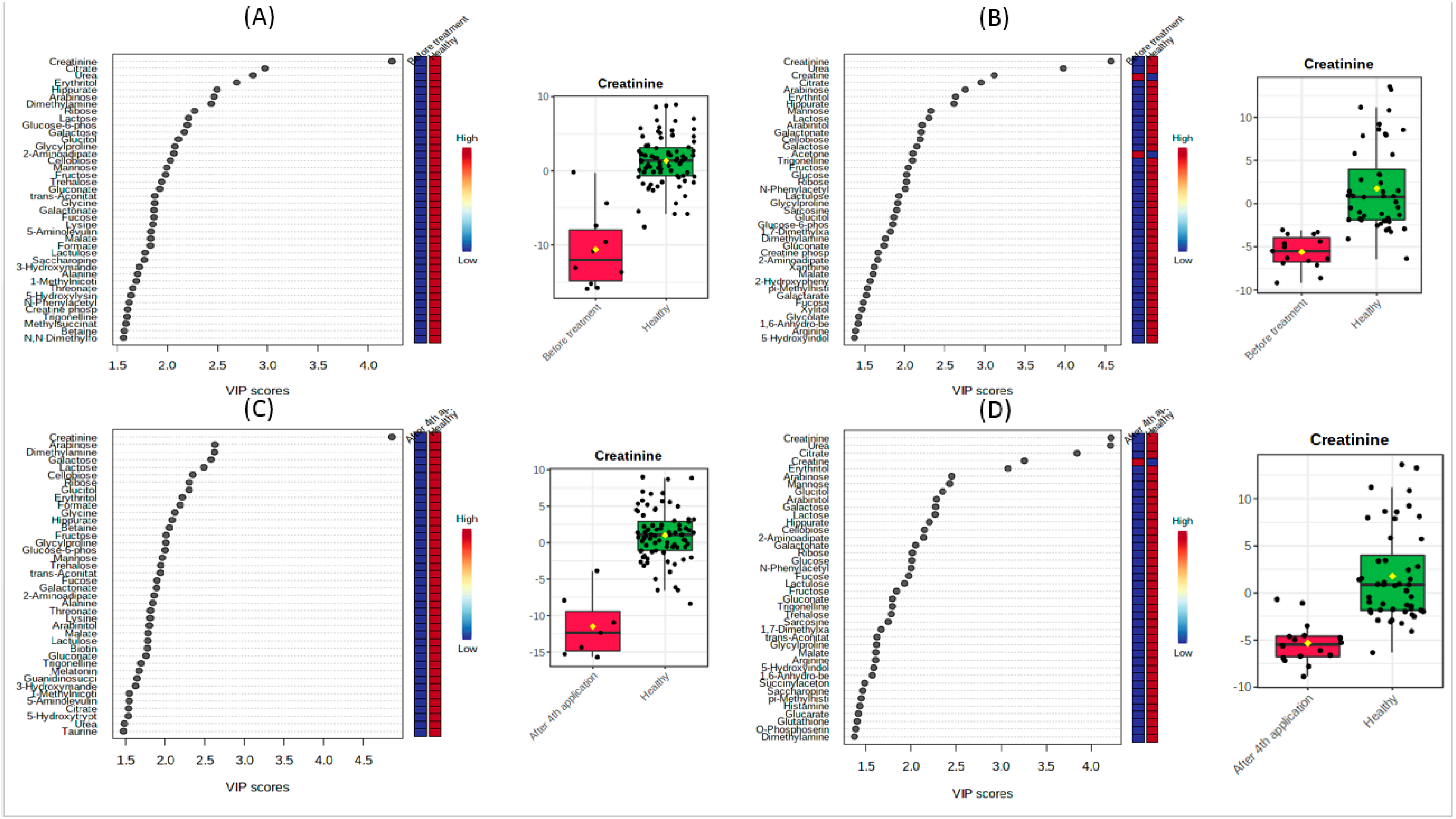

Figure S4. Feature importance plots (MetaboAnalyst PLSDA analysis) represents that Creatinine is the most important metabolite which differentiates between healthy (red column) and SMA patients (blue column), before treatment (A – males, B - females) and after the 4th application (C- males, D- females). This was also confirmed by belonging boxplots. Cohorts were analyzed together.

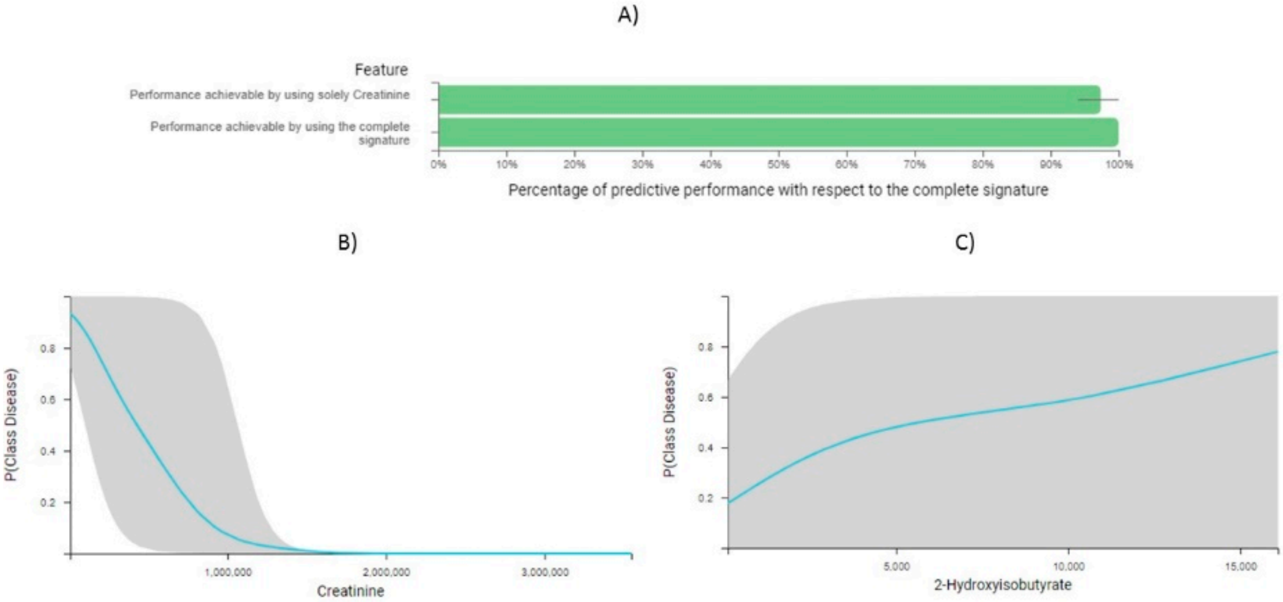

Figure S5. Progressive feature inclusion plot (A) on the predictive performance that can be achieved using only part of the features creatinine (B) and 2-hydroxyisobutyrate (C). Grey lines indicates 95% confindence interval. Decreasing concentration of creatinine and increasing concentration of 2-hydoxyisbutiyrate increase the likelihood of such a sample being classified in SMA group, however, the contribution of the latter is rather ambiguous.

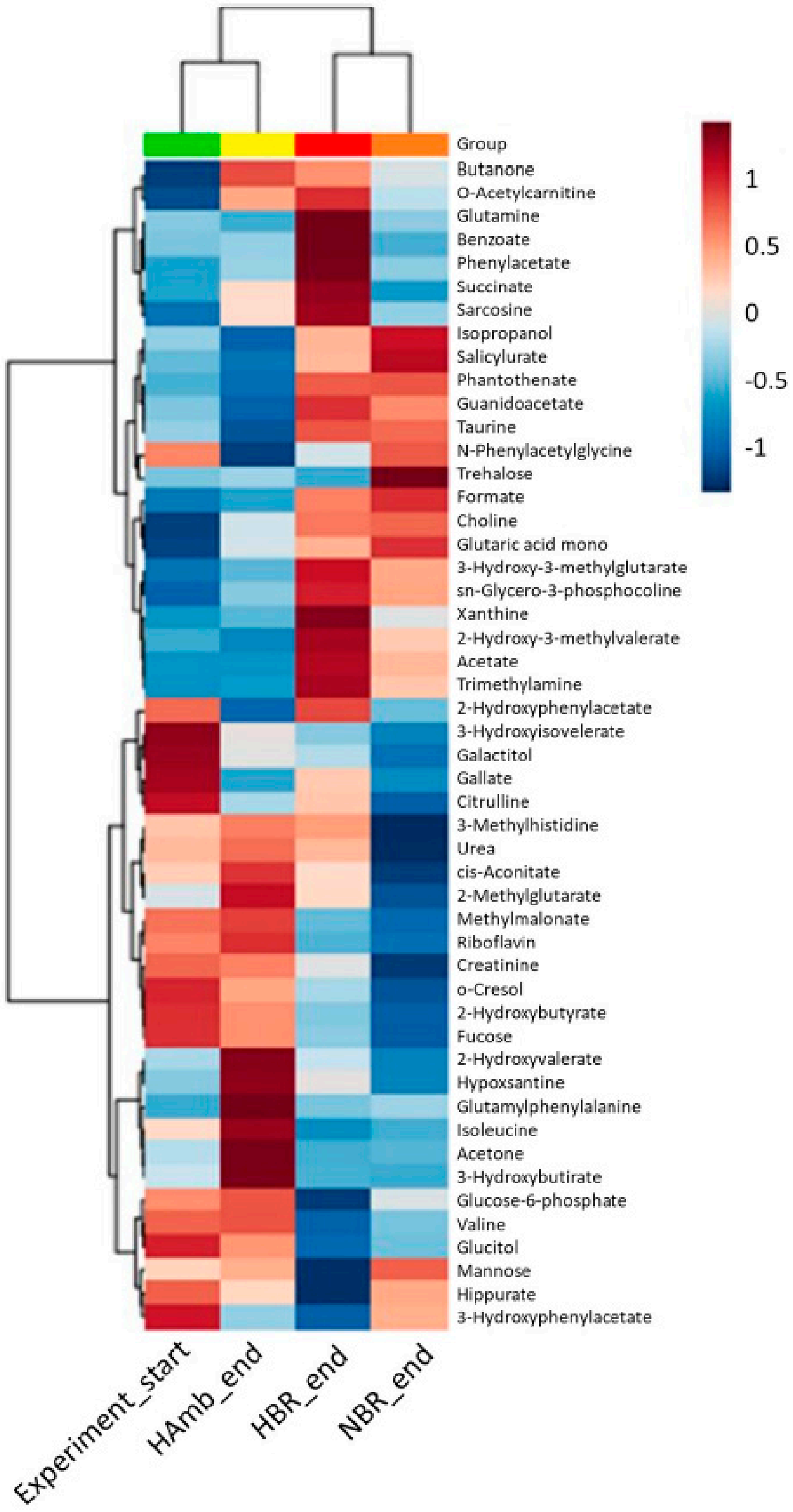

Figure S6: Heatmap of the 50 most important urine metabolites according to AMOVA significance testing constructed using Euclidean distance measure and Ward clustering algorithm. The inset to the left represents the magnitude of z-normalized data. Please note the decreased creatinine concentration in inactivity phenotype (NBR-normoxic bedrest; HBR – Hypoxic bedrest; HAmb- Hypoxic ambulatory).

(Reproduced from Sket et al., 2020. Authors publishing with Frontiers have their articles under the Creative Commons licence CC-BY which permits re-use, distribution and reproduction of material from published articles, provided the original authors and source are credited).

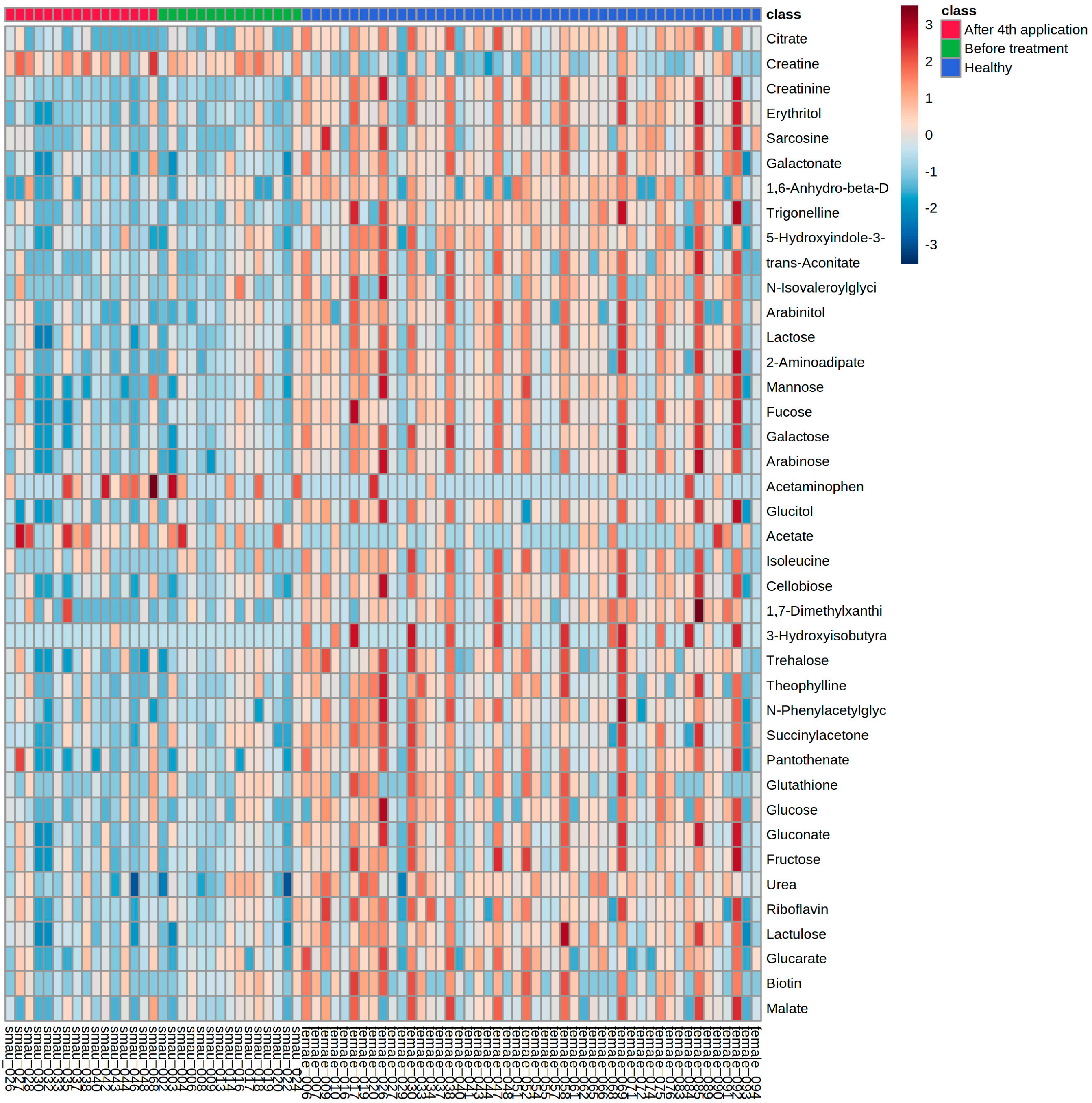

Figure S7: Heatmap representing 40 the most discriminative metabolic features for female SMA patients before and after treatment and matched healthy cohort. Heatmap was generated using PLSDA VIP score with negative values representing decreasing metabolites (blue) and positive values representing increasing metabolites (red).

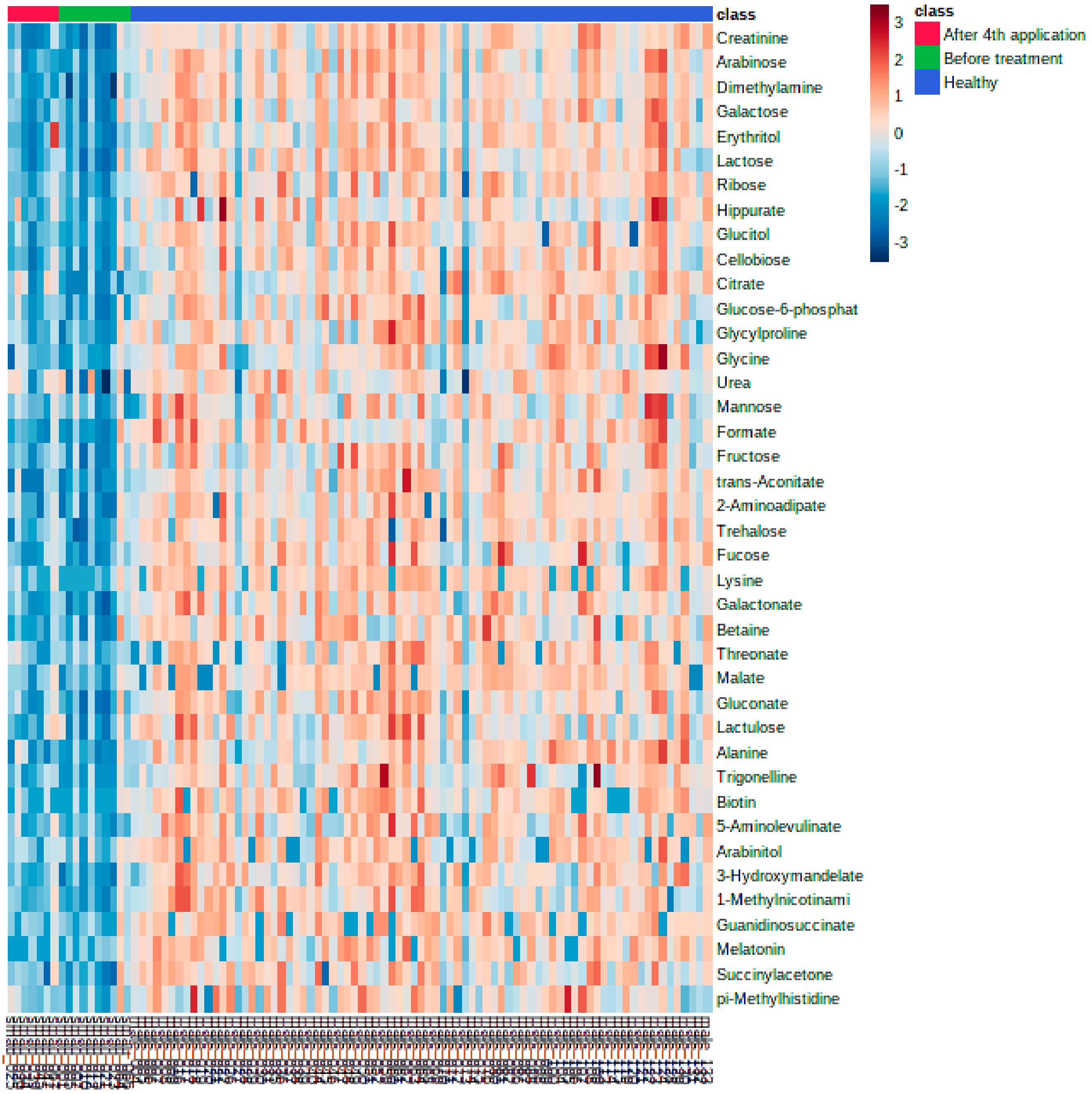

Figure S8: Heatmap representing 40 the most discriminative metabolic features for male SMA patients before and after treatment and matched healthy cohort. Heatmap was generated using PLSDA VIP score with negative values representing decreasing metabolites (blue) and positive values representing increasing metabolites (red).

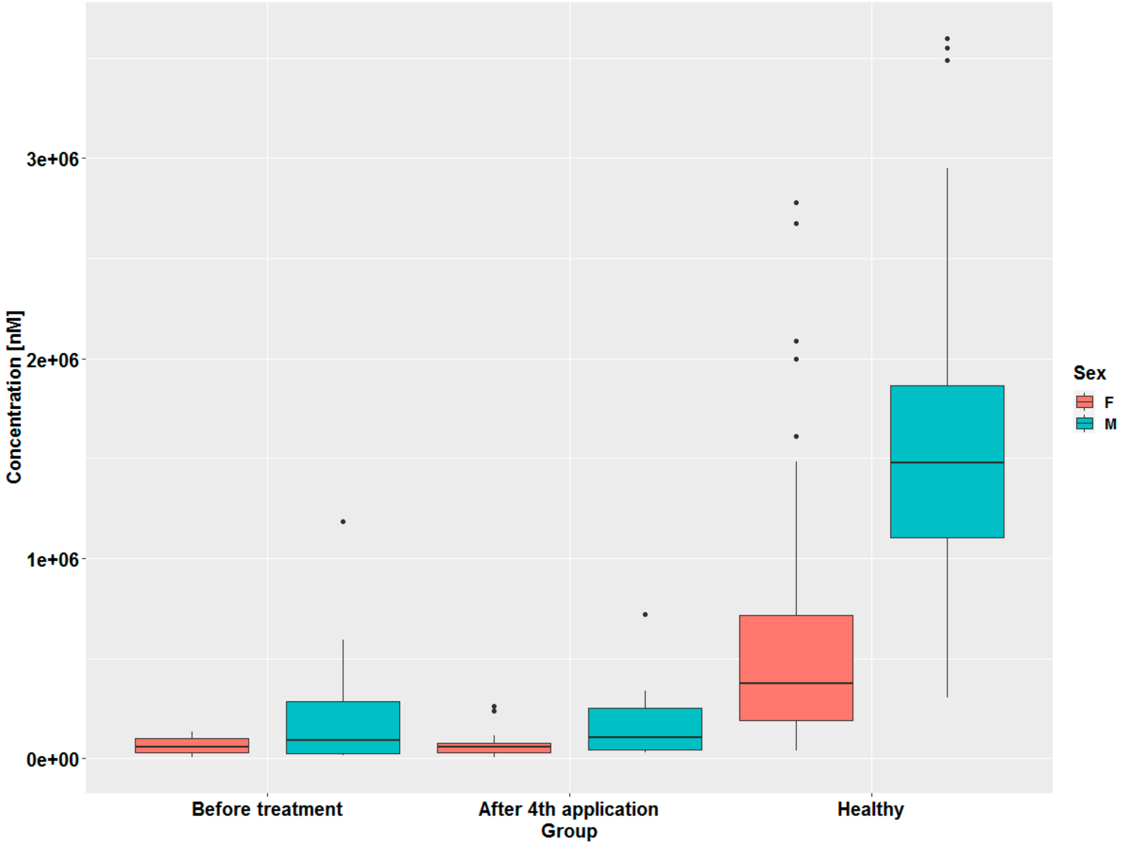

Figure S9: Box-plots representing Creatinine concentrations in all three groups (Before treatment, After 4th application, Healthy) in females and males separately.

Table S1. Descriptive statistics of analyzed cohorts. For SMA cohorts, some data are missing due to disease status.

	SMA males before treatment		males after 4th applica		Healthy males		MA females before treatmen		IA females after 4th applicati		Healthy females	
	Average	SD	Average	SD	Average	SD	Average	SD	Average	SD	Average	SD
Age (years)	9.3	5.1	8.9	5.0	9.6	4.3	8.8	5.5	8.5	5.8	9.4	3.5
Height (cm)	130.9	20.4	132.0	23.7	141.9	24.4	131.3	20.7	123.9	26.0	135.7	19.5
Weight (kg)	28.3	16.4	27.7	14.9	36.2	16.1	26.2	15.2	23.7	14.6	31.5	13.4
BMI (kg/m^2)	17.2	5.2	16.2	3.3	16.9	2.5	16.0	4.9	15.3	1.9	16.3	2.0
n	10		8		77		15		15		48	

Table S2: The number of detected metabolites per sample in the three SMA matrices before and after nusinersen therapy, relative to metabolites detected in urine samples of healthy cohort. SD – one standard deviation.

Sample	Gender	Group	Average	SD
**Urine**	Male	Before	166.3	20.6
		After	170.0	11.0
		Healthy	178.2	10.8
	Female	Before	153.4	30.3
		After	140.4	52.0
		Healthy	154.9	24.0
**Liqour**	Male	Before	44.9	14.5
		After	49.1	11.0
	Female	Before	45.4	12.2
		After	41.1	11.4
**Serum**	Male	Before	40.1	22.7
		After	47.3	24.2
	Female	Before	44.1	26.1
		After	48.1	17.1

Table S3. Performance metrics of the best interpretable model created by JADBIO AutoML platform

Metric	Mean estimate	95% confidence interval
Area under the curve	0.958	[0.873, 1.00]
Average Precision	0.979	[0.908, 1.00]
Accuracy	0.929	[0.826, 0.982]
F1 score	0.863	[0.764, 0.954]
Matthews correlation	0.772	[0.580, 0.905]
Precision	0.79	[0.606, 0.960]
True Positive Rate	0.903	[0.744, 1.00]
Specificity	0.88	[0.783, 0.983]
True positives	0.266	[0.190, 0.340]
True negatives	0.621	[0.531, 0.724]
False positives	0.084	[0.012, 0.157]
False negatives	0.029	[0.00, 0.075]

Instructions for running a model on a local machine

JADBIO allows the user to download a model and run it on a local machine.

To run our model locally, the user must meet the following requirements:

1. Java SE Development Kit version 15 (https://www.oracle.com/java/technologies/javase-jdk15-downloads.html)

2. the Java executor (contained in model.zip - filename: jadbio-1.1.179-model-exe.jar)

3. model (contained in model.zip - filename: jadbio-1.1.164-model.bin)

After installing Java SE JDK, model.zip must be saved somewhere on the local machine. After saving model.zip, provided by the authors, the folder must be extracted (e.g. with WinZip, 7zip). The model must be executed with the command prompt (cmd) (Fig. 1, 2).

Step 1

Using the cd path command (Fig. 1), the user navigates to the same directory (e.g. Folder) that contains the model executor (.jar) and the model (.bin).

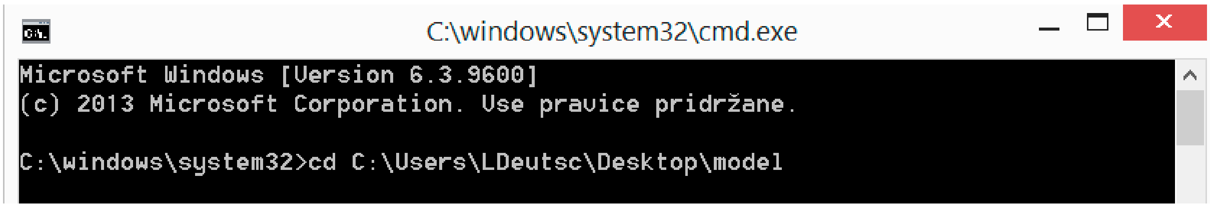

Figure I1: First command to navigate to the folder containing the model. In this case, we used the cd C:\Users\LDeutsc\Desktop\model command because Executor and Model were in the Model folder on the desktop.

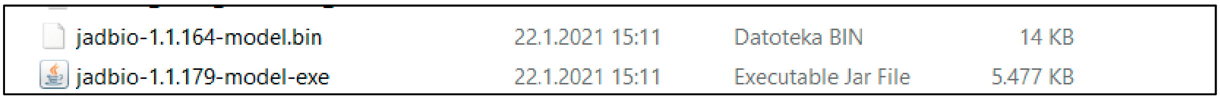

Figure I2: The two files needed for the overview of the model. Both files are contained in the model.zip folder.

Step 2

The next step is to preview the model using the following command:

Java --enable-preview -jar jadbio-1.1.179-model-exe.jar -n jadbio-1.1.164-model.bin

This allows the user to get an overview of the model, key features and information about the analysis (which algorithm was used, version of JADBIO and other information about the model) (Fig. 3).

```
C:\Users\LDeutsc\Desktop\model>java --enable-preview -jar jadbio-1.1.179-model-e
xe.jar -n jadbio-1.1.164-model.bin

Model created by JAD version:
  1.1.164

Model:
  Ridge Logistic Regression Model

Signature variables:
  Creatinine,2-Hydroxyisobutyrate

Analysis info:
  id=10940, title='Training.diseaseVsHealthy.noDSS_Label.extensive', type='CLASS
IFICATION', target='Label', dataset='Training.diseaseVsHealthy.noDSS', dataset_i
d='10661', project='SMA vs. Healthy', metric='AUC', feature_selection=true, inte
rpretable=false, max_vars=25, max_signatures=null
```

Figure I3: Overview of the model.

Step 3

To test the model, the user must prepare data. For the model to work properly, the concentrations must be in nM. The user needs to prepare the dataset as shown in Figure 4 and save it in a .csv document (comma separated values). After preparation, the prepared dataset must be saved in the same directory as the model executor and the model itself.

	A	B	C	D
1	Sample na	Creatinine	2-Hydroxyisobutyrate	
2	female_00	378500	1500	
3	female_00	436300	1400	
4	female_01	185600	400	
5	smau_005	58100	1000	
6	smau_007	128200	1900	
7	smau_008	56500	600	
8				
9				
10				

Figure 4: Prepared dataset.

Step 4

After saving the dataset, the user must use the next command in the terminal:

java --enable-preview -jar jadbio-1.1.179-model-exe.jar -m jadbio-1.1.164-model.bin -i test2.csv -o test2-output.csv

This command runs the model on test data (test2.csv in our case) and creates a new dataset with predictions (test2-outpu.csv) (Fig. 5).

```
C:\Users\LDeutsc\Desktop\model>java --enable-preview -jar jadbio-1.1.179-model-e
xe.jar -m jadbio-1.1.164-model.bin -i test2.csv -o test2-output.csv
Successfully loaded model from jadbio-1.1.164-model.bin
Successfully loaded input dataset from test2.csv
Successfully wrote predictions to test2-output.csv

C:\Users\LDeutsc\Desktop\model>
```

Figure I5. Executing the model and creating the output .csv file with predictions in the same directory.

Step 5

After model execution, the user can check the calculated predictions by opening the .csv file directly by clicking on the created .csv file and opening it in any data analysis program (Excel, Past, R ...). As shown in Fig. 6 the model correctly classifies the data as healthy and as disease. The first column is the same as in the test data created by the user. The second and third columns show the calculated probabilities for the Disease class (second column) and for the Healthy class (third column).

	A	B	C
1	Sample name	Prob (class = Disease)	Prob (class = Healthy)
2	female_006	0.150682351	0.849317649
3	female_009	0.081026818	0.918973182
4	female_010	0.3141834	0.6858166
5	smau_005	0.755516135	0.244483865
6	smau_007	0.780185384	0.219814616
7	smau_008	0.681039375	0.318960625

Figure I6. The newly created .csv file with predictions calculated from test data.
